# Supplementary material for: Investigating pleiotropic effects of statins on ischemic heart disease in the UK Biobank using Mendelian randomisation
Source: eLife. 2020 Aug 25;9:e58567. doi: 10.7554/eLife.58567 (PMC7449694; doi:10.7554/eLife.58567)
Supplement: Supplementary file 1. — (a) SNP-specific estimates for SNPs mimicking effects of statins, PCSK9 inhibitor and ezetimibe on LDL-cholesterol (effect size) in women and men from the UK Biobank, and for comparison estimates for both sexes together from the Global Lipids Genetics Consortium (GLGC) (Willer et al., 2013). (b) SNP-specific estimates for anakinra and tocilizumab SNPs on IL1-Ra (Interleukin 1 Genetics Consortium, 2015) and IL-6 (Swerdlow et al., 2012) respectively. [file elife-58567-supp1.docx]

Table S1: SNP-specific estimates for statin, PCSK9 inhibitor and ezetimibe SNPs on LDL-cholesterol (effect size) in women and men from the UK Biobank, and for comparison estimates for both sexes together from the Global Lipids Genetics Consortium (GLGC) [1].

| Therapy | Sex | Source | SNP | Effect allele | beta | se | p-value | F-statistic |
| --- | --- | --- | --- | --- | --- | --- | --- | --- |
| Statin | Women | UK Biobank | rs12916 | C | 0.0695 | 0.0033 | 3.2E-100 | 452.2 |
|  | Men | UK Biobank |  |  | 0.0527 | 0.0036 | 2.5E-49 | 218.2 |
|  | All | GLGC |  |  | 0.0733 | 0.0038 | 7.8E-78 | na |
| PCSK9 | Women | UK Biobank | rs11206510 | T | 0.0501 | 0.0041 | 2.7E-34 | 149.2 |
| inhibitor | men | UK Biobank |  |  | 0.0454 | 0.0045 | 2.9E-24 | 103.3 |
|  | All | GLGC |  |  | 0.0831 | 0.005 | 2.4E-53 | na |
|  | Women | UK Biobank | rs2149041 | G | 0.0414 | 0.0041 | 8.1E-24 | 101.3 |
|  | Men | UK Biobank |  |  | 0.0439 | 0.0045 | 1.7E-22 | 95.2 |
|  | All | GLGC |  |  | 0.0636 | 0.0049 | 1.4E-35 | na |
|  | Women | UK Biobank | rs7552841 | T | 0.0260 | 0.0034 | 2.7E-14 | 57.9 |
|  | Men | UK Biobank |  |  | 0.0207 | 0.0037 | 3.0E-08 | 30.7 |
|  | All | GLGC |  |  | 0.0368 | 0.0044 | 5.4E-15 | na |
| Ezetimibe | Women | UK Biobank | rs10260606* | C | 0.0407 | 0.0041 | 8.5E-23 | 96.6 |
|  | Men | UK Biobank |  |  | 0.0295 | 0.0045 | 6.9E-11 | 45.2 |
|  | All | GLGC |  |  | 0.0427 | 0.0427 | 3.3E-17 | na |

* in place of rs2073547

Table S2: SNP-specific estimates for anakinra and tocilizumab on IL1-Ra [2] and IL-6 [3] respectively

| Therapy | Target | SNP | Effect allele | beta | se | F-statistic |
| --- | --- | --- | --- | --- | --- | --- |
| Anakinra | IL-1Ra | rs6743376 | C | 0.25 | 0.025 | 100 |
|  |  | rs1542176 | C | 0.18 | 0.025 | 51.8 |
| Tocilizumab | IL-6 | rs7529229 | C | 0.09 | 0.005 | 3600 |

1. Willer CJ, Schmidt EM, Sengupta S, Peloso GM, Gustafsson S, Kanoni S, Ganna A, Chen J, Buchkovich ML, Mora S *et al*: **Discovery and refinement of loci associated with lipid levels**. *Nature genetics* 2013, **45**(11):1274-1283.

2. **Cardiometabolic effects of genetic upregulation of the interleukin 1 receptor antagonist: a Mendelian randomisation analysis**. *Lancet Diabetes Endocrinol* 2015, **3**(4):243-253.

3. Swerdlow DI, Holmes MV, Kuchenbaecker KB, Engmann JE, Shah T, Sofat R, Guo Y, Chung C, Peasey A, Pfister R *et al*: **The interleukin-6 receptor as a target for prevention of coronary heart disease: a mendelian randomisation analysis**. *Lancet (London, England)* 2012, **379**(9822):1214-1224.
